# Insights into Cellular Localization and Environmental Influences on the Toxicity of Marine Fish-Killing Flagellate, *Heterosigma akashiwo*

**DOI:** 10.3390/ijms241210333

**Published:** 2023-06-19

**Authors:** Malihe Mehdizadeh Allaf, Charles G. Trick

**Affiliations:** 1 Mechanics of Active Fluids and Bacterial Physics Laboratory, Department of Civil and Environmental Engineering, Western University, London, ON N6A 3K7, Canada; mmehdiz@uwo.ca; 2 Department of Health and Society, University of Toronto, Toronto, ON M1C 1A4, Canada

**Keywords:** experimental design, *Heterosigma akashiwo*, toxicity, *Saccharomyces cerevisiae*, temperature, salinity, light

## Abstract

*Heterosigma akashiwo* is a unicellular microalga which can cause massive mortality in both wild and cultivated fish worldwide, resulting in substantial economic losses. Environmental parameters such as salinity, light, and temperature showed a significant effect on bloom initiation and the toxicity of *H. akashiwo*. While in previous studies a one-factor-at-a-time (OFAT) approach was utilized, which only changes one variable at a time while keeping others constant, in the current study a more precise and effective design of experiment (DOE) approach, was used to investigate the simultaneous effect of three factors and their interactions. The study employed a central composite design (CCD) to investigate the effect of salinity, light intensity, and temperature on the toxicity, lipid, and protein production of *H. akashiwo*. A yeast cell assay was developed to assess toxicity, which offers rapid and convenient cytotoxicity measurements using a lower volume of samples compared to conventional methods using the whole organism. The obtained results showed that the optimum condition for toxicity of *H. akashiwo* was 25 °C, a salinity of 17.5, and a light intensity of 250 μmol photons m^−2^ s^−1^. The highest amount of lipid and protein was found at 25 °C, a salinity of 30, and a light intensity of 250 μmol photons m^−2^ s^−1^. Consequently, the combination of warm water mixing with lower salinity river input has the potential to enhance *H. akashiwo* toxicity, which aligns with environmental reports that establish a correlation between warm summers and extensive runoff conditions that indicate the greatest concern for aquaculture facilities.

## 1. Introduction

The ichthyotoxic raphidophyte *Heterosigma akashiwo*, a golden–brown unicellular microalga causing harmful algal blooms, has been frequently observed in coastal waters around the world over the past few decades [1,2,3,4]. *H. akashiwo* blooms are sporadic and are responsible for the fatality of cultured and wild fish in different parts of the world such as North America (including Canada and the United States) [4,5,6,7,8], Mexico [9], Japan [10,11], Chile [12], China [13], New Zealand [14], Europe (such as Spain) [15], and Norway [16]. The global loss of resources due to this species is several million dollars each year [17,18]. During a four-month period in 1997 alone, *H. akashiwo* blooms resulted in a greater than $20 million loss of fisheries stock in British Columbia, Canada [6]. 

The toxin and mechanism by which *H. akashiwo* and other ichthyotoxic raphidophytes kill fishes are not clear. So far, four different mechanisms have been hypothesized regarding the fish kills: (1) mucous secretion by *H. akashiwo* resulting in fish asphyxiation by covering fish gills [14], (2) production of brevetoxin-like neurotoxin compounds causing cardiac disorders and/or gill damage [10,19], (3) production of reactive oxygen species (ROS) such as superoxide, hydrogen peroxide, and hydroxyl radicals affecting fish gill [20,21], and (4) production of hemagglutination and hemolysis compounds which causes blood cell lysis [22].

The most routinely employed method to measure the ichthyotoxic effect of marine microalgae in a laboratory is a bioassay, using whole organisms or larvae such as brine shrimp (*Artemia salina*) [23,24,25], Japanese sea bream fish (*Pagrus major*) [10,26], and yellowtail (*Seriola quinqueradiata*) [27]. The mouse bioassay is also widely used worldwide. However, this bioassay is unreliable and ethically questionable [28,29]. On the other hand, each whole-organism bioassay experiment requires prolonged exposure or follow-up periods (72 h or greater) and is prone to errors in evaluation; particularly, high levels of variance [24]. Therefore, finding a rapid, inexpensive, and reliable bioassay model for evaluating the toxicity of harmful algal species is required. This study aims to address this research gap by proposing a novel bioassay model which will be discussed in detail later in this article.

Different environmental factors affect toxin production depending on the HABs taxon [11,30,31,32,33,34]. A direct correlation was detected between environmental factors such as light, temperature and salinity and the growth and toxicity of *H. akashiwo* in different geographical areas [3,10,11,23,24]. For the Japanese strain the highest toxicity was observed at a light intensity of 200 μmol photons m^−2^ s^−1^, and a temperature lower than 25 °C [10]. The highest cellular toxicity of *H. akashiwo* (NWFSC-513) which was isolated from the bloom in 2010 in the Salish Sea, the inland waters of southwestern British Columbia, Canada, and northwestern Washington, USA, was detected at a temperature of 14.7 °C and a salinity of 32 [3]. The bloom of *H. akashiwo* was observed for the first time in Red Sea waters, off the coast of Saudi Arabia in May 2010. It occurred when there was a decrease in salinity (<30) and an increase in temperature (>19 °C) [23]. The results of toxicological assays from the bloom samples presented greater toxicity and hemolytic activity than the batch cultures [23].

Environmental stressors can impact the cellular-level production rate of lipids and proteins, which are essential constituents of cells. While lipids are structural components of cell membranes, modulate cellular activity, and serve as energy storage compounds [35], proteins work in various ways in a cell as structural and functional components

There is growing concern that climate change could affect the fatty acid composition in aquatic ecosystems drastically [36], leading to a disruption of carbon and energy transfer along the lower food chain.

A positive correlation was proposed between hemolytic activity and polyunsaturated fatty acids (PUFAs) [22]. Similar compounds have been isolated and categorized as PUFAs from the raphidophytes *Fibrocapsa japonica* [37] and *Chattonella marina* [32], which may be the primary causative substances in fish mortalities [37].

The induced allelopathic compounds isolated from *H. akashiwo* were categorized as polysaccharide–protein complexes (APPCs) which can inhibit the competitor’s growth by binding to the cell surface [38].

In this study, the effect of environmental factors such as light, temperature, and salinity on the cellular toxicity, lipid and protein content of *H. akashiwo* were studied. To measure the toxicity of *H. akashiwo*, the use of yeast cells (*Saccharomyces cerevisiae*) as a biological cell model was evaluated. Yeast is a commonly used, easy-to-maintain bioassay species, free from ethical concerns but sensitive to a wide array of metabolic and membrane-modulating agents [39,40]. The majority of the research related to operational characteristics of environmental factors has employed a one-factor-at-a-time (OFAT) approach where only one factor or variable is changed at a time while keeping others fixed [41,42]. The problem associated with this approach is that it cannot quantify the interactions of factors to be taken into account, preventing the determination of optimal operating conditions. In this work, a multivariable study of key parameters on the toxicity, lipid and protein production level of *H. akashiwo* was carried out using a designed experiment. A designed experiment is a more accurate approach compared to OFAT when studying the interaction of more than one parameter since the interaction between different parameters is estimated in a systematic way and a larger portion of the factor space is considered. This leads to a more precise estimation of the response [41,42]. Moreover, fewer resources including experiments, time, and materials are required to obtain the essential information when using a design of experiment approach [41,43,44].

## 2. Results

### 2.1. Toxicity Measurement in H. akashiwo

**Figure 1:**
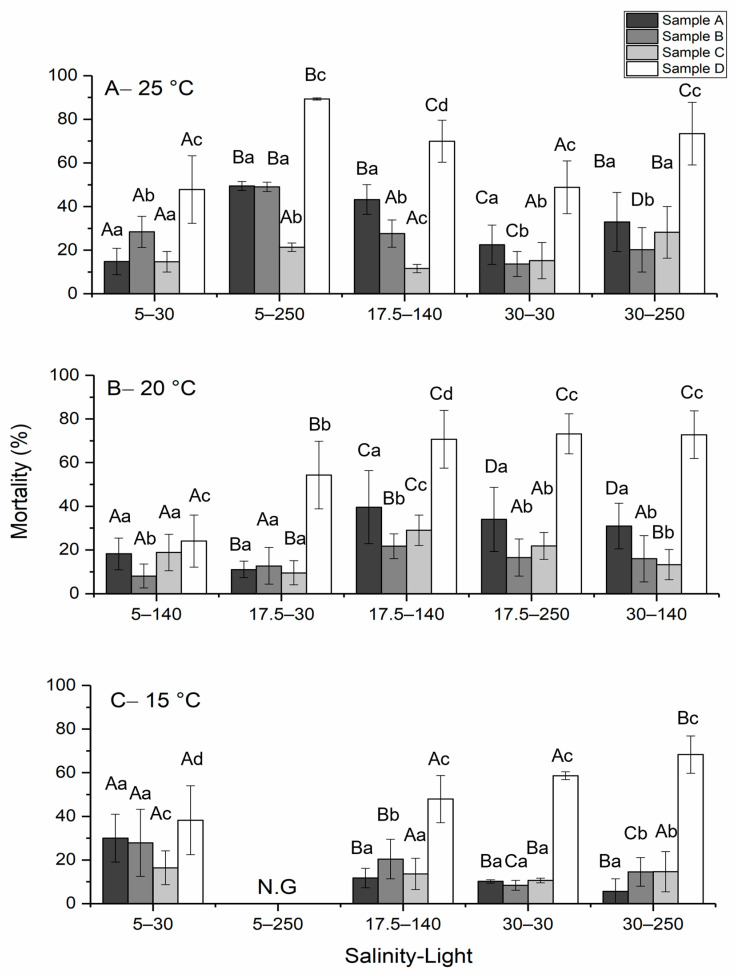
Profiles of toxicity level for *H. akashiwo* at (**A**) 25 °C, (**B**) 20 °C, and (**C**) 15 °C at different salinities and light intensities. The discrete data points are the average of triplicate measurements ± standard deviation (*n* = 3). (N.G., conditions with no growth of cells). The same uppercase letters indicate no significant effect for similar sample treatment under different salinity–light regimes. The same lowercase letters indicate no significant effect among different treatments in the same salinity–light regime. Significance tested at *p* < 0.05 level.

The toxicity of *H. akashiwo* was measured during the stationary phase when the external nitrogen resources are depleted due to phytoplankton uptake [45]. Intact cells (sample A), ruptured cells (sample B) and resuspended, sonicated pellets of *H. akashiwo* (sample D) showed maximum toxicity at 25 °C, a salinity of 5, and a light intensity of 250 μmol photons m^−2^ s^−1^. No growth and therefore no toxin production was observed for cells cultured at 15 °C, a salinity of 5, and a light intensity of 250 μmol photons m^−2^ s^−1^. On the other hand, the maximum level of toxicity for sample C was produced when the cell was cultured at 20 °C, a salinity of 17.5, and a light intensity of 140 μmol photons m^−2^ s^−1^ (Figure 1). A two-way ANOVA followed by the Tukey post-hoc test was used to evaluate differences in toxicity. At the 0.05 level, the population means of treatment (*p*-value = 4.6 × 10^−10^) and temperature (*p*-value = 0.003) were significantly different. In addition, the mortality presented by sample D was significantly different from the rest of the sample treatments. A significant difference was detected between 25 °C and 15 °C (*p*-value = 0.002), while no significant difference was observed at 25 °C and 20 °C (*p*-value = 0.34) and 20 °C and 15 °C (*p*-value = 0.07). 

Among all cell treatments, sample D, which was resuspended and sonicated, showed the highest level of toxicity, in comparison with intact cells and other sample treatments (Figure 1). This suggests that the toxin of *H. akashiwo* is isolated intracellularly and released upon cell rupture.

#### 2.1.1. Determining the Optimum Condition for the Toxicity of *H. akashiwo*

To the best knowledge of authors, in almost all previous studies investigating the effect of environmental factors on the toxicity of *H. akashiwo* a one-factor-at-a-time (OFAT) approach has been employed, in which only one factor is varied at a time while keeping others fixed. The major drawback of the OFAT approach is that it fails to consider any possible interaction between the factors [41]. On the other hand, the design of experiments (DOE) approach, in which several factors vary simultaneously, is more efficient when studying two or more factors [41]. Therefore, to investigate the effect of various environmental factors on the toxicity of *H. akashiwo* using the yeast cell bioassay, the design of experiments (DOE) approach was used. The experimental conditions were chosen based on a central composite design (CCD) (2^3^ three factors at two levels) in combination with the response surface methodology (RSM). The actual values of independent variables and measured responses are presented in Table 1.

#### 2.1.2. Response Surface Model (RSM) Validation 

The complete data set for the toxicity effect of *H. akashiwo* for samples A, C, and D were fitted with a quadratic model as described in Equation (1) and a 2-factor interaction (2FI) model for sample B. The resulting model parameters and experimental input are shown in Table 2. The F-values for samples A, B, C, and D are 3.48, 3.10, 3.43, and 6.40, respectively, which are higher than the critical values, thus indicating the significance of the model. The *p*-value was used to determine the significance of each parameter coefficient. A smaller *p*-value means the coefficient has a higher significance. Temperature and salinity showed a great effect on *H. akashiwo* toxicity. The effect of temperature was significant in samples A, B, and D while salinity showed a significant effect on the toxicity of sample D (Table 2). None of the environmental factors showed any significant effect on sample C. The interaction of salinity and temperature, the interaction of temperature and light, as well as the quadratic effects of salinity, had also significant effects on the toxicity of *H. akashiwo*. To confirm the goodness-of-fit of the models, the coefficient of determination, R^2^ and Adj. R^2^ were used. R^2^ was 0.80, 0.63, 0.79, and 0.88 and Adj. R^2^ was 0.57, 0.43, 0.56, and 0.74 for samples A, B, C, and D, respectively. Adequate precision is used to measure the signal-to-noise ratio. A ratio greater than 4 is desirable. The obtained ratio for samples A, B, C, and D were 5.64, 6.89, 6.02, and 9.46, respectively, indicating an adequate signal. These models can be used to navigate the design space.

Based on the selected significant variables, the final equation in terms of actual factors for each mortality can be calculated using the following equations:

Mortality (A) = −53.3 + 6.47 × Temperature + 0.02 × Temperature × Light

Mortality (B) = + 5.38 + 1.15 × Salinity + 0.01 × Temperature × Light

Mortality (D) = −115.78 + 8.18 × Salinity + 10.77 × Temperature − 0.21 × Salinity × Temperature + 0.02 × Temperature × Light − 0.01 × Salinity^2^

#### 2.1.3. Combined Effect of Salinity, Light, and Temperature on *H. akashiwo* Toxicity

**Figure 2:**
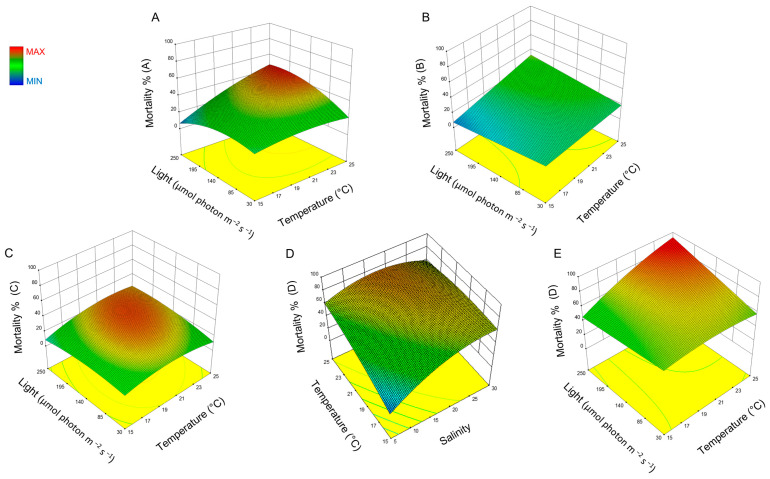
Surface plots of the combined effect of salinity, light, and temperature on the toxicity of *H. akashiwo*. (**A**) mortality for sample A, (**B**) mortality for sample B, (**C**) mortality for sample C, and (**D**,**E**) mortality for sample D.

Response surface methodology (RSM) was used to study the interaction effect of these three factors on the toxicity of *H. akashiwo* and the resulting plots are presented in Figure 2. The combined effect of light and temperature on the toxicity effect of *H. akashiwo* on yeast mortality is illustrated in Figure 2A–C,E. Figure 2D indicates the combined effect of temperature and salinity on this microalga. The mortality of yeast cells, when combined with different fractions of *H. akashiwo*, is a function of temperature, light, and salinity. The surface plots indicate that an optimum is present within the observed design space with respect to salinity and light, and increasing the temperature appears to increase yeast mortality over the observed design space.

#### 2.1.4. Response Optimization and Model Validation

Based on the model, numerical optimization was used to determine the optimal combination of process parameters to estimate the toxicity of *H. akashiwo*. The optimal condition for *H. akashiwo* to produce the highest amount of toxin was 25 °C, a salinity of 17.5, and a light intensity of 250 μmol photons m^−2^ s^−1^, warm and fresher water, and high light. To validate the applicability of this RSM model, confirming experiments were performed around the estimated optimal conditions. The measured and predicted results are presented in Table 3. Comparing the predicted values with measured values with a *t*-test at a 95% confidence interval showed no significant difference between these two sets of data.

The proposed RSM model can be used as a useful tool to predict the toxicity level of *H. akashiwo*. To the best knowledge of the authors, this is the first attempt to model the toxicity production response of *H. akashiwo* under the combined effect of the three environmental factors and their interactions simultaneously.

### 2.2. Lipid and Protein Measurement in H. akashiwo

**Figure 3:**
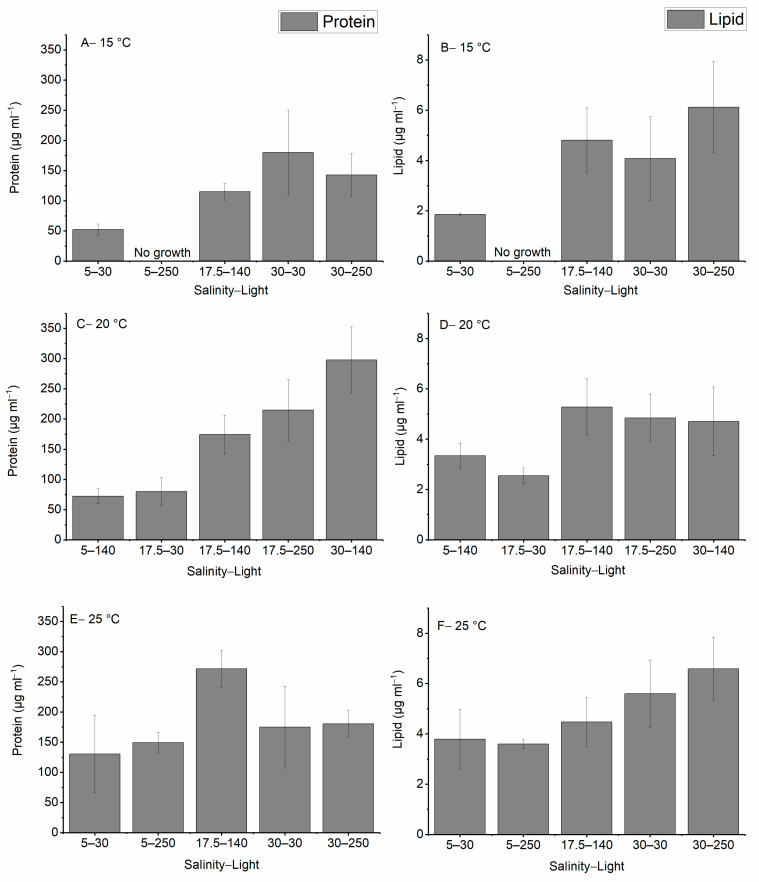
Total amount of protein and lipid produced in *H. akashiwo* under different salinities, light intensities and temperatures (**A**–**F**). The discrete data points are the average of triplicate measurements ± standard deviation (*n* = 3).

In this study, the amount of lipid and protein were measured under different environmental stress conditions and the results are illustrated in Figure 3. The maximum production of protein was observed when the culture was grown at 20 °C, a salinity of 30, and a light intensity of 140 μmol photons m^−2^ s^−1^, and the highest amount of lipid was produced at 25 °C, a salinity of 30, and a light intensity of 250 μmol photons m^−2^ s^−1^. No growth was observed when the cells were cultured at 15 °C, a salinity of 5, and a light intensity of 250 μmol photons m^−2^ s^−1^. In all temperature treatments, the lowest salinity produced the lowest amount of protein and lipid while increasing the temperature, salinity, and light improved the production of both lipid and protein.

Increasing the salinity level while keeping light intensity constant enhanced the production of lipid and protein. For instance, the protein production increased from 72.3 ± 12.5 to 298.0 ± 54.6 as the salinity increased from 5 to 30 at a light intensity of 140 μmol photons m^−2^ s^−1^ at 20 °C.

Significant differences were detected between groups for protein content at 20 and 25 °C and lipid at all treatment temperatures (*p*-value < 0.05).

#### 2.2.1. Determining the Optimum Condition for the Maximum Lipid and Protein Production of *H. akashiwo*

To determine the optimum condition for lipid and protein in *H. akashiwo*, a design of experiment (DOE) was used to investigate the effect of different factors such as salinity, light intensity, and temperature, and their interaction on the response. The experimental data for the measured responses under the aforementioned factors were chosen based on a central composite design and are presented in Table 4. All experiments were completed in triplicates and average values ± standard deviations are presented in Table 4.

#### 2.2.2. Response Surface Model (RSM) Validation

The models for both protein and lipid production in *H. akashiwo* were fitted linearly and the resulting model parameters are displayed in Table 5. The F-values for lipids and proteins are 7.11 and 5.12, respectively. These F-values imply the models are significant. 

In addition, the *p*-values less than < 0.05 is considered significant for each parameter coefficient as well. The small *p*-value (<0.05) for lipids and proteins (0.0039 and 0.0134, respectively) emphasizes the significance of the model. The results indicate that salinity and temperature had a significant effect, Table 5. The goodness-of-fit of each model was approved by the coefficient of determination R^2^ and adjusted determination coefficient Adj.R^2^, Table 5. Based on the selected significant variables, the final equations for protein and lipid production in terms of actual factors are:

Total protein = −77.78 + 4.31 × Salinity + 7.67 × Temperature

Total lipid = −0.96 + 0.12 × Salinity

**Figure 4:**
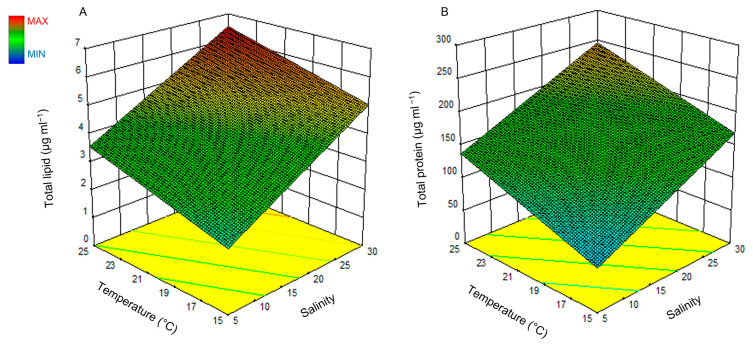
Surface plot of the combined effect of salinity and temperature on lipid (**A**) and protein (**B**) production of *H. akashiwo*.

The response surface plots for both lipid and protein are illustrated in Figure 4, where Figure 4A presents the effect of light and temperature on lipid production and Figure 4B presents the effect of the same factors on the protein production of *H. akashiwo*.

Increasing the temperature and salinity (Figure 4A,B) increases lipid and protein production linearly. However, light did not show any significant effect on lipid and protein production (*p*-value > 0.05). The highest amount of lipid and protein based on the measured ranges for salinity, light, and temperature were predicted to be obtained at 25 °C, a salinity of 30, and a light intensity of 250 μmol photons m^−2^ s^−1^. The model predicted a maximum lipid production value of 6.86 ± 1.13 μg mL^−1^ and a protein production value of 251.5 ± 53.43 μg mL^−1^. A lipid production value of 6.59 ± 1.26 μg mL^−1^ and a protein production of 180.38 ± 21.82 μg mL^−1^ were obtained experimentally which show a very good agreement with the predicted results. *t*-test at a 95% confidence interval did not show a significant difference between the predicted and experimental values.

### 2.3. Relationship between Growth, Yield, and Cell Permeability with Toxicity, Lipid, and Protein Production

**Figure 5:**
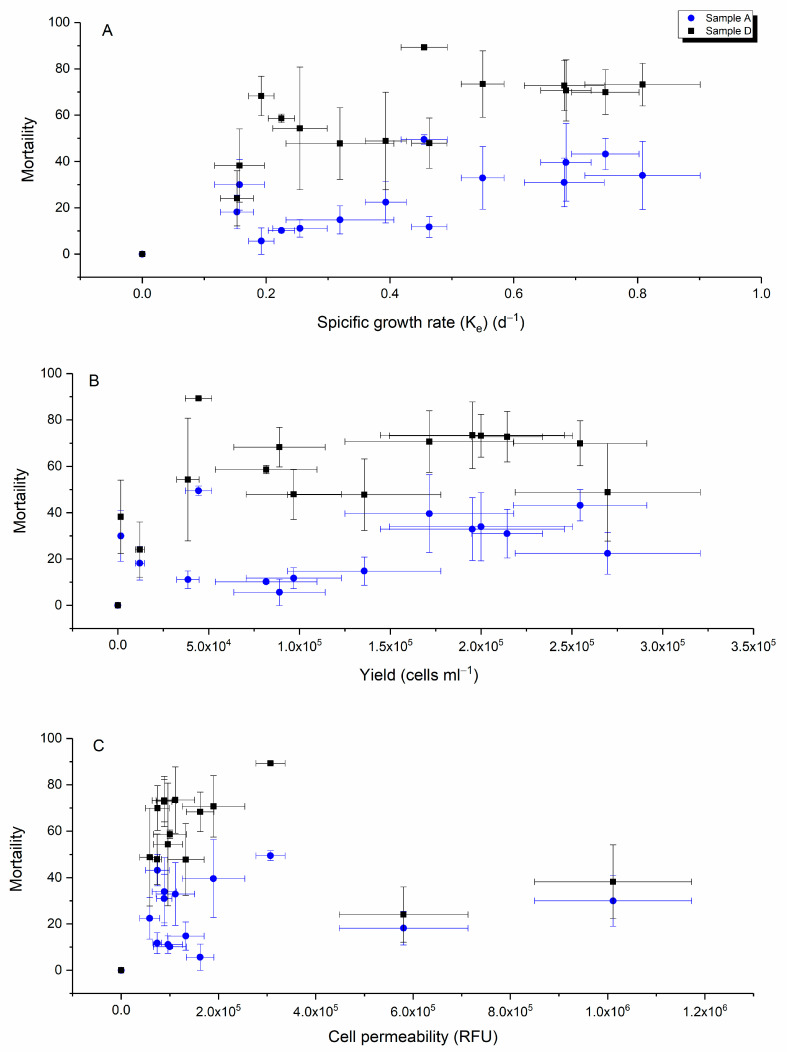
Correlation data between the mortality of yeast cells (%) with *H. akashiwo* toxin and specific growth rate (k_e_) (**A**), yield (**B**) and cell permeability (**C**) (*n* = 3).

Correlating the specific growth rate, yield and cell permeability [46] with the toxicity effect of *H. akashiwo* for intact cells (sample A) and resuspended pellets (sample D) showed the specific growth rate was inversely related to toxicity for both intact and lysed cells, (Figure 5A). In addition, for the same cell biomass, the toxicity level in sample D was greater than in sample A, suggesting that the toxin of *H. akashiwo* is located in cell cytosol or attached to membranes and rupturing the cells helped to release the toxin.

A weak positive relationship was detected between cell permeability and the mortality of yeast cells (Pearson’s r = 0.44), (Figure 5C). While Ikeda et al. [3] proposed that cell permeability was a proxy of *H. akashiwo* toxicity, a comparison of the yeast toxicity with cell permeability indicates otherwise (Figure 5C). Here, highly permeable cells expressed the same as the lower levels of toxicity and cells with the lowest permeability ranged equally from non-toxic to toxic given our present understanding that the toxic metabolites are released on lysis. The cell permeability hypothesis seems logical but ultimately unsubstantiated.

**Figure 6:**
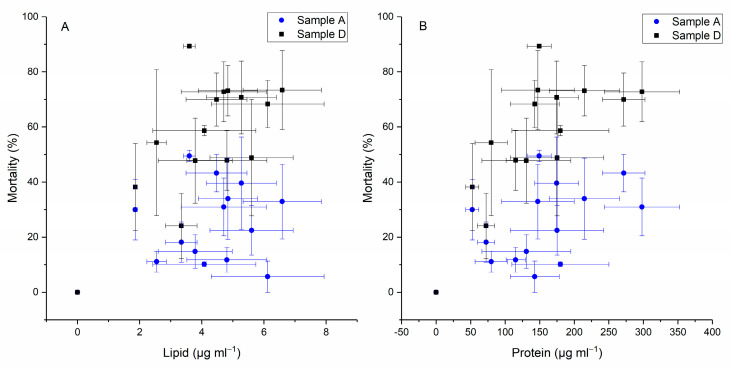
Correlation data between toxicity level and lipid (**A**) and protein (**B**) content of *H. akashiwo*.

As can be seen in the plots in Figure 6, as the cellular toxicity increased, the total amount of protein and lipid production by *H. akashiwo* improved gradually in cells. A strong correlation was not detected between lipid and protein production with the mortality of yeast cells. However, lysing the cell membrane and using the intracellular fraction of *H. akashiwo* (sample D) showed an enhanced liberation of toxin(s) from *H. akashiwo* and a better correlation to cell composition of lipid and protein relative to the intact cells (sample A) (Pearson’s r = 0.7).

A second prevalent hypothesis is that phytotoxins, in this case, individual toxins, are produced based on changes in cell elemental or biochemical stoichiometry [47]. This has been proposed for *H. akashiwo* [38] or *P. parvum* [48] (both fish-killing species). Here, the weak correlation between the cell composition of lipids and proteins and the toxicity of intact cells (sample A) (Figure 6) would argue against the hypothesized stoichiometry model.

**Figure 7:**
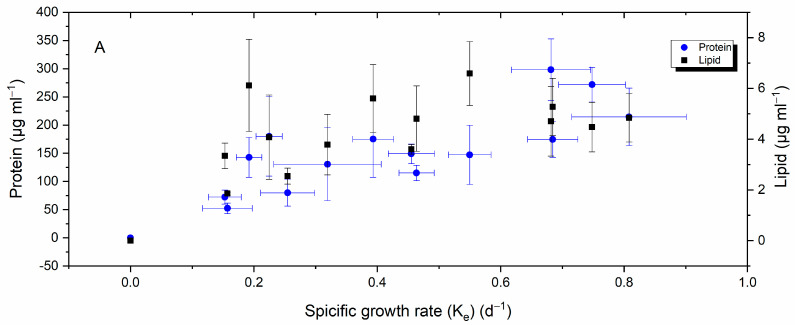

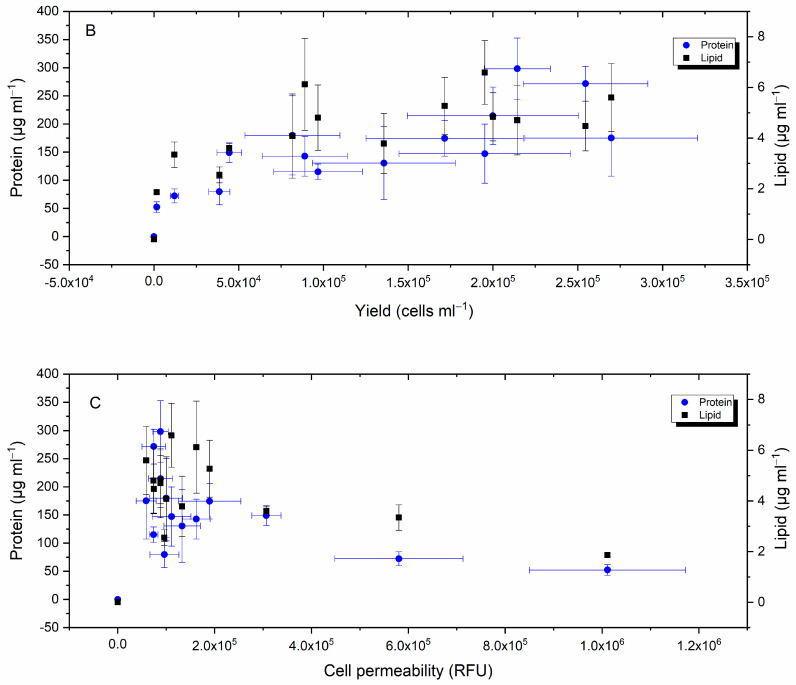
Relationship between *H. akashiwo* growth rate (**A**), yield (**B**), cellular permeability (**C**), and total lipid and protein contents.

Inspection of plots representing the relationship between *H. akashiwo* growth rate, yield, and total lipid and protein content in Figure 7A,B reveals that when yield and the specific growth rate increased, so did protein production and lipid; however, protein production showed a stronger correlation (Pearson’s r > 0.8) compared to lipid (Pearson’s r = 0.6). A negative weak correlation was detected between lipid and protein and cell permeability (Figure 7C).

## 3. Discussion

The results demonstrate two things: First, yeast cells can be used as an appropriate proxy to measure *H. akashiwo* toxicity, offering benefits such as shorter measurement time, smaller volume, and reduced resources; second, the design of experiment (DOE) approach could predict a better understanding of the interaction between multiple stressor factors on *H. akashiwo* under realistic environmental conditions. This will allow us to predict the impact of various environmental parameters on the occurrence and degree of toxicity of *H. akashiwo*, which is crucial to the environment, aquaculture facilities, and public health.

### 3.1. Toxicity Measurement in H. akashiwo

Measuring the level of toxicity in *H. akashiwo* is a complicated process, as the research community has not accepted a single measurable criterion. The common acceptance is that blooms of *H. akashiwo* when encountering caged fish or embayments of fish will quickly, and effectively, kill off the fish leaving floating carcasses. Necropsies of terminated fish reveal the fish have a combination of skin lesions, mucus on the gill surface, and symptoms of asphyxiation [14]. *H. akashiwo* cells are also embedded in the lesions and gill mucus but there is a cause-and-effect debate. *H. akashiwo* as the most abundant phytoplankter would accumulate at these locations after the fact and may not be the causative agent. The debate continues.

There are attributes of *H. akashiwo* that are shared by other fish-killing flagellates and generally absent in non-fish-killing flagellates. For example, *H. akashiwo* has a pronounced ability to produce and excrete ROS (reactive oxygen species) [20,21], produces copious glycoproteins [38] and hemolytic agents [22], and there is a persistent report of a “brevetoxin”-like compound [10,19]. Brevetoxins (PbTxs) are a group of polyether lipid-soluble toxins produced by a marine dinoflagellate known as *Karenia brevis* [49]. Brevetoxin compounds are based on two different ladder frames, PbTx-2 (brevetoxin B) and PbTx-1 (brevetoxin A), which lead to gill damage or cardiac disorders due to binding to and persistent activation of voltage-sensitive sodium channels in cell membranes [19,49,50,51,52].

Early work by Twiner et al. [53,54] identified a brevetoxin-like compound. The putative toxin was a polyether, with a mass of 850–1000 daltons, was water soluble and had a neurotoxic potential in cell models. This compound was not constitutively produced by the cell but expressed at higher levels when growth slowed, and cells were ever energetically limited (by iron limitation, for example, when photosynthesis and the electron system were impaired). The key difference with the well-established brevetoxin was that the compound did not impair Na^+^ transport but rather Ca^+2^ in cell line assessments, and the compound was most effective when ROS products were present in the medium.

Without the ability to measure the toxin, alternate approaches have been taken. Unique processes that correlate to the production of “toxicity” have been employed: hemolytic activity, gill cell degeneration, cell wall permeability, brine shrimp, neurological damage, and developmental damage to zebrafish embryos. There is support and criticism of the use of each surrogate test and we search for a more universal assay. Here we evaluated the use of a simple yeast toxicity bioassay which is a sensitive indicator of the toxin produced by *H. akashiwo*. The results identified the maximum yeast cells mortality at the highest extreme temperature in this experiment—25 °C, followed by 20 °C—which is in agreement with previous studies in which unialgal cultures of *H. akashiwo* isolated from the Salish Sea, USA [3], and the Seto Inland Sea, Japan [10] were used. Using gill cell lines, the average toxicity for unialgal cultures was detected at 24.4 °C and 27.8 °C; however, in contaminated samples with diatom the highest toxicity was observed at 14.7 °C [3]. The Japanese strain of *H. akashiwo* revealed the highest toxicity at 20 °C, using 5 to 6 month-old juvenile red sea bream (*Pagrus major*) for toxicity measurement [10]. Based on these data, it is clear that warmer temperatures enhance the toxicity effect of *H. akashiwo* on yeast cells. 

Light is another major environmental parameter with the ability to affect the growth and toxicity of *H. akashiwo* [10,15]. Our findings showed that increasing the light intensity to 250 μmol photons m^−2^ s^−1^ enhanced the toxicity of *H. akashiwo* cells as well as yeast mortality (Figure 2). The mortality of the red sea bream (*Pagrus major*) increased dramatically at a high light intensity after they were exposed to *H. akashiwo*, while decreasing in the dark and at a low light intensity [10]. Similar results were observed for yellowtails (*Seriola quinqueradiata*) after they were exposed to *C. marina*. Their mortality significantly increased in light, while it decreased in the dark [55]. Ling and Trick [22] suggested that the hemolytic activity of *H. akashiwo*, as a proxy for measuring toxicity, is light-dependent and the highest hemolytic activity was detected at a light intensity of 100 μmol photons m^−2^ s^−1^. Therefore, light intensity appears to play a crucial role in regulating the toxicity of various species of raphidophytes; however, the fish-kill mechanism by light is not well understood and further investigations are required. This suggests that toxin production may be stimulated by high light intensity [22]. Our results showed that the interaction effect of light and temperature was significant in the mortality of yeast cells when they were exposed to an intracellular fraction of *H. akashiwo* (sample D). However, light, as an individual factor, did not show a significant effect on the mortality of yeast cells (Table 2).

Another reported factor with a positive impact on the ichthyotoxicity of *H. akashiwo* was salinity. It has been reported that a decrease in salinity level could increase the toxicity level of *H. akashiwo* [3,11,56]. In a Japanese strain, the highest toxicity was observed at a salinity of 20 after culturing for 10 days [11]. The toxicity of an American strain was reported to increase as the salinity decreased to below 20 [56]. Ikeda et al. [3] stated that the highest toxicity in uncontaminated cultures was observed when the salinity reduced from 20 to 10 for an American strain of *H. akashiwo* as well. These results are in agreement with the results obtained in this study and the low salinity presented the highest amount of toxicity. Salinity had a significant effect on the mortality of yeast cells as an individual factor and also when it interacted with temperature for the intracellular fraction sample of *H. akashiwo* (Table 2). This highlights that little is known about the multiple stressors’ effect on the toxicity of *H. akashiwo*. Our results found evidence for the interaction effect of multiple environmental stressors on the toxicity of *H. akashiwo*.

Another promising finding was that the model predicted that sample D, the intracellular fraction (ultrasonic ruptured cell suspension) from *H. akashiwo* cells, would produce potent mortality in yeast cells compared to other treatment samples with the same cell density. This agrees with OFAT data as well. Based on the obtained data, the toxin(s) are released upon cell damage. Therefore, it is crucial to ensure the qualities of all sonicated suspensions are similar in terms of cell lysis.

Previously, a strong positive correlation was reported between cytotoxicity and cellular permeability of the American strain of *H. akashiwo* [3]. However, we did not detect a strong correlation between these two parameters (sample D, Pearson’s r > 0.45) (Figure 5C). A possible reason for different observations could be linked to the fact that Ikeda et al. [3] used a rainbow trout gill cell line (rTgill-W1) to measure toxicity while we employed yeast cells with a more complex cell wall.

### 3.2. Lipid and Protein Measurement in H. akashiwo

Lipid and protein are two main components with varying percentages and control major functions in any cell including *H. akashiwo*. The isolated hemolytic and allelopathic compounds from raphidophytes, as proxies for toxicity measurement, were categorized as lipid and protein, respectively [22,23,32,37,38]. Van de Waal et al. [47] reported that the toxins produced by phytoplankton are stoichiometrically diverse, ranging from N-rich to C-based cellular elemental ratios. The carbon, nitrogen, and phosphorous ratios affect and reflect the content of major biochemicals including proteins and lipids. 

In this study, we measured the lipid and protein content of the cells using the Nile Red assay and Bradford assay colorimetric methods, respectively. We identified that the highest amount of lipid and protein with the same cell density was produced at the maximum temperature, salinity, and light intensity used in this study (Figure 3 and Figure 4). Lipid production was a function of salinity; however, protein production was a function of salinity and temperature. No interaction effect between the aforementioned environmental factors was observed for these two compounds. The light intensity did not show a significant effect on the production of lipid and protein in this study, but it is an essential parameter to convert CO_2_ and nutrients to organic compounds such as lipid and protein in primary producers [47].

The results of this study showed a weak correlation between the cell composition of lipid and protein in intact cells versus yeast mortality (Figure 6, sample A). It was observed that when cells were centrifuged and sonicated, their intracellular fraction showed a better correlation. This implies enhanced liberation of the toxin(s) from *H. akashiwo* by lysing the cell membrane.

The hemolytic compounds isolated from other raphidophytes such as *Fibrocapsa japonica* [37] and *C. marina* [32] have been identified as polyunsaturated fatty acids (PUFAs) either present as free acids, phospholipids or glycolipids and may be the primary causative substances in fish mortalities [37]. *H. akashiwo* hemolytic agents have not been isolated yet but it was suggested that these hemolytic agents may be PUFAs and released upon cell lysis [22]. A strong correlation was detected between temperature and a reduction in the proportion of n-3 long-chain polyunsaturated fatty acid (LC-PUFA) and an increase in omega-6 fatty acid and saturated fatty acid in different species of phytoplankton [36]. Further investigation is required to elucidate the lipid compositions in *H. akashiwo*.

Under nutrient-limited conditions, additional energy and newly synthesized organic carbon cannot be used by the phytoplankton cells for their growth; instead, they can be converted into C-rich molecules such as lipid [47]. Nitrogen deficiency and temperature cause a high-level cellular accumulation of neutral lipids in *H. akashiwo* [35]. The net neutral lipid production per cell in *H. akashiwo* was 30% higher when cells were cultured at 25 °C, 330 μM NaNO_3_ in comparison with cultures grown at 20 °C, 880 μM NaNO_3_. Our findings showed that the lipid production of *H. akashiwo* was enhanced as the temperature increased but it was not significant. The highest level of lipid was produced when cells were in their stationary phase and nitrogen depletion happened [35,45].

High-molecular-weight allelochemicals produced from *H. akashiwo* were identified as polysaccharide–protein complexes (APPCs) [38]. In a mixed culture of *H. akashiwo* and diatom *Skeletonema costatum*, which is a major competitor of *H. akashiwo*, the produced APPCs functioned as glycoproteins and bound to the cell surface of *Skeletonema costatum* and induced an allelopathic effect which inhibited the growth of this diatom [38]. Based on our results, a strong correlation was not detected between protein production and mortality of the yeast cells. However, the intracellular fraction of *H. akashiwo* showed a better correlation compared to intact samples. These findings emphasize that damaging the cell membrane improved the toxicity effect of *H. akashiwo*.

## 4. Materials and Methods

### 4.1. Cultures

The non-axenic strain of *H. akashiwo* (NWFSC-513), isolated in 2010 from Clam Bay, WA, USA, was used in this study. The stock cultures were maintained in f/2 (minus Si) medium [57], in 250 mL Erlenmeyer flasks at 20 ± 1 °C and under a continuous light intensity of 80 ± 5 μmol photons m^−2^ s^−1^. Experimental algal samples were prepared from exponentially growing cultures and grown at different salinities. Salinity is a dimensionless parameter that quantifies the ratio of the mass of dissolved salts to the mass of seawater. Media with different ranges of salinity were prepared by adding varying mass amounts of NaCl (Sigma-Aldrich, Oakville, ON, Canada) to artificial seawater (ESAW) [57]. The experimental flasks were diluted to 10,000 cells mL^−1^ and incubated in a Panasonic Climatic Chamber equipped with fluorescence lamps and forced air circulation. The photosynthetic photon flux density was measured using a Quantum Scalar Laboratory (QSL) sensor (Biospherical Inc., San Diego, CA, USA). All the treatments were performed in triplicate.

*Saccharomyces cerevisiae*, Living, Tube (Merlan Scientific Ltd., Toronto, ON, Canada) was grown at room temperature in YPD plate medium (1% yeast extract, 2% peptone, 2% dextrose, 2% agar) (all *w*/*v*) for 15 ± 1 h.

### 4.2. Algal Samples Preparation for Toxicity Assay

**Figure 8:**
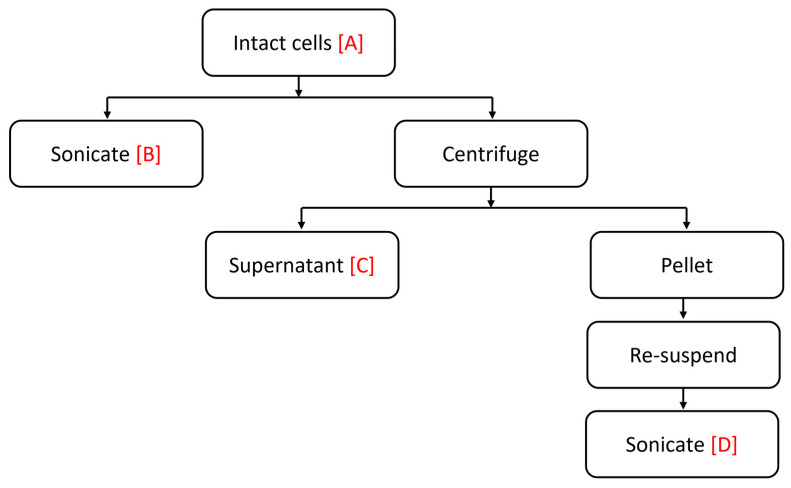
Summary of algal sample preparation.

In order to measure toxicity, algal samples were given enough time to grow and reach their stationary phase to ensure the cells were nutrient depleted and as a result increases the level of toxicity [45]. Cells were collected and fractionated to assess the location of the toxin (Figure 8) as follows:

Sample A: intact cells, viable cells, and extracellular material.

Sample B: ultrasonic ruptured cell suspension was obtained by sonicating 5 mL of the culture suspension in an ice bath with a continuous output power of 9 for 1 min with a Virsonic 100 Ultrasonic Cell Disrupter (VirTis Company, Gardiner, NY, USA).

Sample C: culture supernatant, was prepared by centrifuging a 10 mL sample, using 15 mL falcon centrifuge tubes in a Beckman GH-3.8/GH-3.8A swing-out rotor (Beckman Coulter, Fullerton, CA, USA) at 500× *g* for 10 min at 4 °C.

Sample D: resuspended, sonicated pellets, were resuspended in artificial seawater (ASW) and sonicated for 1 min with a continuous output power of 9.

The algal sample preparation procedure is outlined in Figure 1.

### 4.3. Toxicity Measurement

Samples were prepared with a cell ratio of 5 *H. akashiwo* cells to 1 yeast cell. Samples were incubated at room temperature for 3 h. A Turner Designs PhytoCyt flow cytometer (Sunnyvale, CA, USA) with associated CFlow^®^ Plus software, version 1.0.227.5 was used to measure the number of cells and fluorescence intensity. A dual-fluorescence scatter plot of fluorescence dye versus chl_a_ was used. Each plot was divided into four quadrants that separated the stained and nonstained cells in the upper and lower quadrant, respectively.

In order to measure the cell integrity and the percentage of dead cells, 1.5 μM SYTOX^®^ Green (Life Technologies, Eugene, OR, USA) was added to each sample 15 min before cell measurement. SYTOX^®^ Green is a high-affinity nucleic acid stain that only penetrates into dead cells and cells with compromised permeability, and binds to DNA and fluorescence using a 488 ex (nm) laser and 523 em (nm) detector [58,59].

The toxicity effect of each algal sample was presented as the percentage of yeast mortality using Equation (1):

(1) $$\%\;Mortality=\frac{\#\;Dead\;cells}{\left(\#\;Dead\;cells+\#\;Live\;cells\right)}\times 100$$

The fluorochrome SYTOX^®^ Green was employed to estimate the degree of cell membrane permeability of yeast during treatments. The highest toxicity is expressed in samples when the cells are the most permeable, as indicated by the greatest level of SYTOX^®^ Green fluorescence per cell. In addition, the SYTOX^®^ Green fluorescence does not overlap with the autofluorescence of chlorophyll [60].

### 4.4. Sample Preparation to Measure Total Proteins and Lipids

To determine the total amount of protein and neutral lipid, 10 mL of each sample adjusted at 1 × 10^3^ cell mL^−1^ was collected during the stationary phase and centrifuged at 500× *g* for 10 min at 4 °C. The obtained pellets were frozen at −20 °C till the experiment was performed.

### 4.5. Determination of Total Proteins

Extracellular, high-molecular-weight allelochemicals produced by *H. akashiwo* arepolysaccharide–protein complexes with selectively inhibitory effect on multispecies phytoplankton community [38]. To estimate the total proteins, the Bradford assay [61] was used. It provides a very reproducible and rapid method to determine the concentration of solubilized protein [61] using an acidic solution of Coomassie^®^ Brilliant Blue dye (Bio-Rad protein assay, dye reagent concentrate #51558A). The protein solution was prepared by thawing the frozen cell pellets at room temperature and diluting each sample in 300 μL ultra-pure water. An aliquot of 10 μL of protein solution was mixed with 250 μL of the Bradford dye in a 96-well plate. The plate was incubated for 5 min at room temperature for colour development and the total protein content was measured at 595 nm using a Multiskan GO microplate spectrophotometer (Thermo Scientific, Waltham, MA, USA). Total protein concentration was estimated using a Bovine Serum Albumin (BSA) standard curve, in a linear range of 0–500 μg mL^−1^ (R^2^ > 0.99). All samples were prepared in triplicates.

### 4.6. Determination of Neutral Lipids

To determine the amount of neutral lipid in the samples, a Nile Red assay in a 96-well plate was used. Nile red (9-(Diethylamino)-5H benzo [∞] phenoxa-zin-5-one) is a red phenoxazone and lipid-soluble dye which can be used to detect neutral lipids in vivo. Although a very poorly fluorescent dye in aqueous solutions, it is quite photo-stable and highly fluorescent in non-polar hydrophobic environments [62,63]. For this purpose, 100 μL of the aliquot sample was pipetted into a black-sided clear-bottomed plate and 100 μL of the Nile Red dye (1 μg mL^−1^—Sigma) solution, prepared in 50% DMSO (CalBioChem), was added. To read the fluorescence intensity the plate was kept in the Gemini XPS microplate reader for 10 min at 40 °C. Fluorescence intensities were measured using 530 and 570 nm excitation/emission wavelengths, respectively. All assay samples were performed in triplicate.

### 4.7. Experimental Design

In order to calculate the response pattern and determine the optimal combination of salinity, light intensity, and temperature leading to maximum toxicity, lipid, and protein production of *H. akashiwo*, a central composite design (CCD) with three factors was used. Before expanding the design to a CCD, an initial two-level full factorial design was performed, while the highest and lowest level of each factor was chosen based on the highest and lowest growth rate of *H. akashiwo* in the preliminary experiments (data not shown). The results showed significant curvature and confirmed the significant effect of all three parameters (data not shown). The un-coded values for each parameter were as follows [low star point, center point, high star point]: Salinity [5, 17.5, 30], temperature [15, 20, 25 °C], and light irradiance [30, 140, 250 μmol photons m^−2^ s^−1^]. Design Expert 10.0.3.1 (Stat-Ease, Inc., Minneapolis, MS, USA) was used to develop the experimental design and resulted in 14 conditions. All conditions were tested in triplicate, including three center points. The resulting 51 conditions (8 × 3 factorial + 6 × 3 augmented + 3 × 3 center points) were fully randomized.

### 4.8. Statistical Analysis

The linear regression analysis was used to fit the experimental data with a second-order model (Equation (2)).

(2) $$Y=\beta_{0}+\sum_{i=1}^{3}\beta_{i}x_{i}+\sum_{i=1}^{3}\beta_{ii}x_{i}^{2}+\sum_{1\leq i\leq j}^{3}\beta_{ij}x_{i}x_{j}+\varepsilon$$

The experimental data were analyzed using the statistical Design Expert 10.0.3.1. software and analysis of variance (ANOVA) with an α of 0.05 was conducted to test the significance of each term. To evaluate the adequacy of the fitted model, the normal probability plots, R^2^ and adjusted R^2^, and lack-of-fit coefficient were considered. Then the optimal condition to obtain the maximum toxicity, lipid, and protein content of *H. akashiwo* was determined using the numerical optimization via Design Expert 10.0.3.1. In order to validate the model and optimization results, confirmation experiments were performed around the predicted optimum points. list the authority that provided approval and the corresponding ethical approval code.

## 5. Conclusions

The toxicity of *H. akashiwo* is not constitutive but related to the conditions of growth. Scientific combinations of multiple environmental factors including salinity, temperature and light on the optimum conditions lead to maximum cellular toxicity. The obtained data showed that the DOE approach can be used as an appropriate method to evaluate the effect of various parameters such as temperature, salinity and light on the toxicity of *H. akashiwo*.

The conditions at which *H. akashiwo* reaches the highest level of toxicity included 25 °C, a salinity of 17.5, and a light intensity of 250 μmol photons m^−2^ s^−1^, which represent the warm water mixing with lower salinity river input. These findings are consistent with environmental reports that correlate warm summers with extensive runoff as the conditions of greatest concern to aquaculture facilities [64].

The results also revealed that the yeast bioassay was a highly sensitive indicator of the toxicity of *H. akashiwo*. The small volume of sample required and the ability to perform the test in a short period of time makes this method convenient and saves resources and money. In addition, a large number of samples, which are easy to prepare, can be tested simultaneously. Using different fractions of *H. akashiwo* cells to measure toxicity suggested that the *H. akashiwo* toxin is located in the cellular compartment of cells and rupturing cells with a centrifuge and sonicating the resuspended pellets released more toxin and increased the mortality level of yeast cells. However, there is no standard analytic or method of toxicity to compare the yeast model to fully establish its suitability as a toxicity proxy.

Surprisingly, based on the prevalence of the stoichiometry model of toxin regulation [47], toxicity correlated poorly with cell composition. The warmer water temperature with high salinity input lead to maximum cell composition of protein and lipid.

## Figures and Tables

**Table 1:** The toxicity of *H. akashiwo* grown under conditions for central composite design (*n* = 3 ± SD).

S.	T. (°C)	L. (μmol Photons m^−2^ s^−1^)	Mortality (%)			
			S.A	S.B	S.C	S.D
5	15	30	30.0 ± 11	27.9 ± 15.3	16.4 ± 7.7	38.2 ± 15.8
5	25	30	14. 8 ± 6.1	28.4 ± 7.1	14.7 ± 4.7	47.8 ± 15.5
5	20	140	18.2 ± 7.3	8.1 ± 5.5	18.8 ± 8.3	24.1 ± 11.9
5	15	250	N.G.	N.G.	N.G.	N.G.
5	25	250	49.5 ± 2.04	49.02 ± 2.2	21.3 ± 1.9	89.3 ± 0.5
17.5	20	30	11.1 ± 3.8	12.7 ± 8.4	9.5 ± 5.5	54.3 ± 15.5
17.5	15	140	11.7 ± 4.5	20.4 ± 9.04	13.6 ± 7.2	47.9 ± 10.6
17.5	20	140	41.0 ± 18.2	21.1 ± 5.9	29.0 ± 6.3	72.2 ± 14.7
17.5	20	140	40.2 ± 20.2	21.5 ± 6.7	29.7 ± 7.5	73 ± 17.6
17.5	20	140	39.7 ± 15.7	20.5 ± 4.4	30.0 ± 8.2	71.6 ± 12.5
17.5	20	140	33.7 ± 17.9	21.6 ± 7.6	29.8 ± 8.4	66.9 ± 18.1
17.5	20	140	43.2 ± 11.7	23.6 ± 3.8	26.6 ± 4.4	69.9 ± 3.2
17.5	25	140	43.2 ± 6.8	27.6 ± 6.3	11.6 ± 1.9	69.9 ± 9.6
17.5	20	250	34.0 ± 14.7	16.5 ± 8.4	21.8 ± 6.1	73.2 ± 9.2
30	15	30	10.2 ± 0.8	8.4 ± 2.2	10.6 ± 1	58.6 ± 1.8
30	25	30	22.5 ± 9	13.7 ± 5.8	15.2 ± 8.4	48.8 ± 12.1
30	20	140	30.9 ± 10.5	16.0 ± 10.5	13.3 ± 6.9	72.8 ± 10.9
30	15	250	5.6 ± 4.7	14.5 ± 6.6	14.6 ± 9.2	68.3 ± 8.5
30	25	250	32.9 ± 13.5	20.2 ± 10.2	28.2 ± 11.8	73.4 ± 14.3

Parameters: S. = Salinity; T. = temperature; L. = Light; Experimental input: S.A = Sample A; S.B = Sample B; S.C = Sample C; S.D = Sample D; N.G. = No growth.

**Table 2:** Analysis of variance of fitted model for different levels of toxicity in *H. akashiwo*.

Source	M_1_ (%)	Model	S_2_ (A)	T_3_ (B)	L_4_ (C)	AB	AC	BC	A^2^	B^2^	C^2^
Remark	Sample A	S_5_.	N-S_6_.	S.	N-S.	N-S.	N-S.	S.	N-S.	N-S.	N-S.
	Sample B	S.	N-S.	S.	N-S.	N-S.	N-S.	S.			
	Sample C	S.	N-S.	N-S.	N-S.	N-S.	N-S.	N-S.	N-S.	N-S.	N-S.
	Sample D	S.	S.	S.	N-S.	S.	N-S.	S.	S.	N-S.	N-S.
F-value	Sample A	3.48	0.11	11.19	1.13	0.03	1.8 × 10^−3^	7.99	1.43	0.52	2.34
	Sample B	3.10	2.63	7.30	0.14	2.97	0.79	4.77			
	Sample C	3.43	0.34	3.84	1.14	7.8 × 10^−3^	2.67	3.84	1.32	4.58	1.62
	Sample D	6.40	12.42	11.16	2.63	11.07	0.99	9.25	5.14	0.52	1 × 10^−4^
*p*-value	Sample A	0.0466	0.75	0.01	0.32	0.86	0.97	0.02	0.27	0.49	0.16
	Sample B	0.0498	0.13	0.02	0.72	0.11	0.39	0.05			
	Sample C	0.0484	0.58	0.09	0.32	0.93	0.14	0.09	0.28	0.06	0.24
	Sample D	0.0078	0.008	0.01	0.14	0.01	0.35	0.02	0.05	0.49	0.99
R^2^	Sample A	0.80									
	Sample B	0.63									
	Sample C	0.79									
	Sample D	0.88									
Adj. R^2^	Sample A	0.57									
	Sample B	0.43									
	Sample C	0.56									
	Sample D	0.74									
Adeq. precision	Sample A	5.64									
	Sample B	6.89									
	Sample C	6.02									
	Sample D	9.46									

_1_ = Mortality, _2_ = Salinity, _3_ = Temperature, _4_ = Light, _5_ = Significant, _6_ = Not significant.

**Table 3:** Optimal conditions and model validation for toxicity effect of *H. akashiwo* on the yeast cells viability.

S	T	L	Mortality (%)							
			Sample A		Sample B		Sample C		Sample D	
			Pre	Exp	Pre	Exp	Pre	Exp	Pre	Exp
17.31	25	246	47.7 ± 9.9	40.6 ± 10.2	32.7 ± 7.9	26.2 ± 6.8	24 ± 5.8	19.5 ± 8.3	92.5 ± 11	83.4 ± 2.4
17.13	25	244	47.8 ± 9.9	40.4 ± 3.9	33 ± 7.9	26.8 ± 1.7	24 ± 5.8	14.4 ± 10.6	92.1 ± 11	82.6 ± 1.5
17.18	25	250	47.5 ± 9.9	46.2 ± 10.8	33.3 ± 7.9	27.9 ± 10.6	23.8 ± 5.8	24.3 ± 9.1	93.1 ± 11	82.6 ± 2

S (Salinity), T (temperature (°C)), L (Light (μmol photons m^−2^ s^−1^), Pre (predicted), Exp (Experimental).

**Table 4:** Lipid and protein production in *H. akashiwo* under conditions for central composite design (*n* = 3).

Salinity	Temperature (°C)	Light (μmol Photons m^−2^ s^−1^)	Protein (μg mL^−1^)	Lipid (μg mL^−1^)
5	15	30	52.3 ± 9.4	1.9 ± 0.1
5	25	30	130.6 ± 64.4	3.8 ± 1.2
5	20	140	72.3 ± 12.5	3.3 ± 0.5
5	15	250	N.G.	
5	25	250	149.2 ± 14.4	3.6 ± 0.2
17.5	20	30	79.7 ± 23.3	2.5 ± 0.3
17.5	15	140	115.1 ± 13.9	4.8 ± 1.3
17.5	20	140	174.0 ± 29.8	5.2 ± 0.2
17.5	20	140	176.6 ± 34.9	5.6 ± 1.7
17.5	20	140	178.0 ± 31	5.1 ± 1
17.5	20	140	158.1 ± 23.4	5.0 ± 0.9
17.5	20	140	186.1 ± 39.5	5.6 ± 1.2
17.5	25	140	271.6 ± 30.8	4.5 ± 1
17.5	20	250	214.6 ± 51	4.8 ± 0.9
30	15	30	179.9 ± 70.3	4.1 ± 1.7
30	25	30	175.1 ± 67.6	5.6 ± 1.3
30	20	140	298.0 ± 54.6	4.7 ± 1.4
30	15	250	142.8 ± 35.4	6.1 ± 1.8
30	25	250	180.4 ± 21.8	6.6 ± 1.3

N.G. = No growth.

**Table 5:** Analysis of variance of fitted model for lipid and protein production in *H. akashiwo*.

Source		Model	Salinity	Temperature (°C)	Light (μmol Photons m^−2^ s^−1^)
Remark	Lipid Protein	Significant Significant	Significant Significant	Not significant Significant	Not significant Not significant
F-value	Lipid Protein	7.11 5.12	16.45 10.16	4.03 5.15	0.84 0.046
*p*-value	Lipid Protein	0.001 0.01	0.001 0.01	0.06 0.04	0.38 0.83
R^2^	Lipid Protein	0.60 0.52			
Adj-R^2^	Lipid Protein	0.52 0.42			
Adeq-precision	Lipid Protein	8.6 7			

## Data Availability

The data is available upon request.
